# Supplementary material for: Telehealth satisfaction among patients with chronic diseases: a cross-sectional analysis
Source: PeerJ. 2025 Apr 25;13:e19245. doi: 10.7717/peerj.19245 (PMC12036575; doi:10.7717/peerj.19245)
Supplement: Supplemental Information 2 [file peerj-13-19245-s002.docx]

**Characteristics of studied patients:**

|  |
| --- |
| Age:………….years |
| Gender:  Male  Female |
| Education level:  Preparatory  Secondary  Bachelor |
| Marital status:  Married  Unmarried |
| Income level:  Insufficient  Sufficient  Sufficient and save |
| Having children:  Yes  No |
| Easy to contact with IT at hospital  Always  Sometimes  Never |
| Continuous training about using tele-health  Yes  No |
| Residence  Far from hospital  Near to hospital |
| Employment:  Work  Not-work |

**Tool2:**

**10-item Telehealth Satisfaction Scale:**

|  | *Excellent* | *Good* | *Fair* | *Poor* |
| --- | --- | --- | --- | --- |
| *How satisfied were you with:* | | | | |
| *1 The voice quality of the equipment* |  |  |  |  |
| *2 The visual quality of the equipment* |  |  |  |  |
| *3 Your personal comfort in using the* |  |  |  |  |
| *Telehealth system* |  |  |  |  |
| *4 The ease of getting to the telehealth department* |  |  |  |  |
| *5 The length of time with the Memory Clinic team* |  |  |  |  |
| *6 The explanation of your treatment by the Memory Clinic team* |  |  |  |  |
| *7 The thoroughness, carefulness and skillfulness of the Memory Clinic team* |  |  |  |  |
| *8 The courtesy, respect, sensitivity, and friendliness of the Memory Clinic team* |  |  |  |  |
| *9 How well your privacy was respected* |  |  |  |  |
| *10 How well the staff answered your questions about the equipment* |  |  |  |  |
